# Young People’s Online Help-Seeking and Mental Health Difficulties: Systematic Narrative Review

**DOI:** 10.2196/13873

**Published:** 2019-11-19

**Authors:** Claudette Pretorius, Derek Chambers, David Coyle

**Affiliations:** 1 School of Computer Science University College Dublin Dublin Ireland; 2 Connecting for Life Health Service Executive Cork Ireland

**Keywords:** internet, help-seeking behavior, youth, mental health, online behavior, self-determination theory, systematic review

## Abstract

**Background:**

Young people frequently make use of the internet as part of their day-to-day activities, and this has extended to their help-seeking behavior. Offline help-seeking is known to be impeded by a number of barriers including stigma and a preference for self-reliance. Online help-seeking may offer an additional domain where young people can seek help for mental health difficulties without being encumbered by these same barriers.

**Objective:**

The objective of this systematic literature review was to examine young peoples’ online help-seeking behaviors for mental health concerns. It aimed to summarize young peoples’ experiences and identify benefits and limitations of online help-seeking for this age group. It also examined the theoretical perspectives that have been applied to understand online help-seeking.

**Methods:**

A systematic review of peer-reviewed research papers from the following major electronic databases was conducted: PsycINFO, Cumulative Index of Nursing and Allied Health Literature, PubMed, Cochrane Library, Association for Computing Machinery Digital Library, and Institute of Electrical and Electronics Engineers Xplore. The Preferred Reporting Items for Systematic Reviews and Meta-Analyses guidelines were followed. The search was conducted in August 2017. The narrative synthesis approach to reviews was used to analyze the existing evidence to answer the review questions.

**Results:**

Overall, 28 studies were included. The most common method of data collection was through the use of surveys. Study quality was moderate to strong. Text-based query via an internet search engine was the most commonly identified help-seeking approach. Social media, government or charity websites, live chat, instant messaging, and online communities were also used. Key benefits included anonymity and privacy, immediacy, ease of access, inclusivity, the ability to connect with others and share experiences, and a greater sense of control over the help-seeking journey. Online help-seeking has the potential to meet the needs of those with a preference for self-reliance or act as a gateway to further help-seeking. Barriers to help-seeking included a lack of mental health literacy, concerns about privacy and confidentiality, and uncertainty about the trustworthiness of online resources. Until now, there has been limited development and use of theoretical models to guide research on online help-seeking.

**Conclusions:**

Approaches to improving help-seeking by young people should consider the role of the internet and online resources as an adjunct to offline help-seeking. This review identifies opportunities and challenges in this space. It highlights the limited use of theoretical frameworks to help conceptualize online help-seeking. *Self-determination theory* and the *help-seeking model* provide promising starting points for the development of online help-seeking theories. This review discusses the use of these theories to conceptualize online help-seeking and identify key motivations and tensions that may arise when young people seek help online.

## Introduction

### Background

Mental health is an important health concern for young people around the world, with the World Health Organization estimating that 10% to 20% of young people experience mental health disorders [1]. It is estimated that 50% of all adult mental disorders start in adolescence [2]. Yet, most young people are reluctant to seek help from formal mental health services [3-5]. The help-seeking process is difficult; it is complicated by personal and contextual factors, such as access, stigma, and mental health literacy [3,6,7]. It then becomes critically important to find alternative methods in which to target and assist young people who are not receiving help.

**Figure 1:**
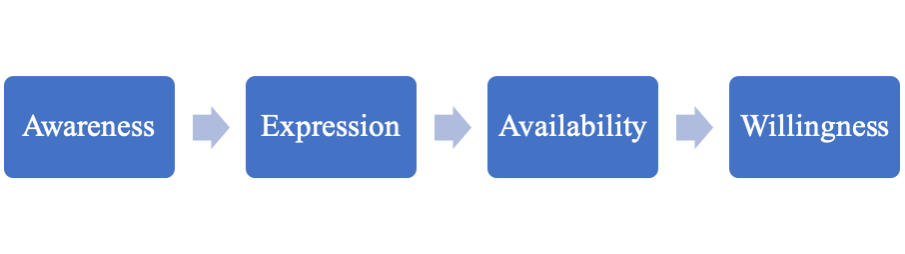
Rickwood's Help-seeking model.

Mental health help-seeking has been defined as “...an adaptive coping process that is the attempt to obtain external assistance to deal with a mental health concern” (p. 180) [8]. That external assistance can be from a wide array of sources. Rickwood et al [3] propose a conceptual model specifically taking into account the needs of young people (Figure 1). They describe help-seeking as a process that involves the following: (1) becoming aware of symptoms and making the appraisal that assistance might be required; (2) expressing the symptoms that they are experiencing and that they are in need of help or support; (3) the person should then be aware of sources of help that are available and accessible to them; and (4) the final step that depends on the willingness of the help-seeker to disclose their difficulties to the selected, available source [3].

Two main types of help-seeking sources have been identified: formal and informal. Formal help-seeking can be understood as seeking assistance from any professional who has a recognized and legitimate role in providing support. Informal help-seeking is understood as pursuing assistance from informal social supports with whom the individual may or may not share a personal relationship [3]. Research shows that most young people have a preference for self-reliance when experiencing personal and emotional concerns and are more likely to make use of informal help sources than formal help sources, when and if they do reach out [3].

Most recently, computer-mediated technologies have begun to influence the help-seeking process. The internet offers another pathway to access care and help when young people are experiencing mental health concerns. Young people use the internet as their main source of information for all of their daily needs; accordingly, this logically extends to accessing information regarding their physical and mental health [9-11]. Various formal online services are readily available, as are informal resources such as discussion boards and social media [12]. Information gained from these sources could facilitate the help-seeking process to the next stage and could influence the way in which individuals form their help-seeking attitudes. The internet also offers unique benefits in the form of anonymity, access, and user control that can sometimes interfere with the offline help-seeking process [13]. The availability of high-quality mental health information and online resources could have a significant impact on the health outcomes of a young person [14].

### Objectives

Although the potential benefits of online mental health resources have been acknowledged, there are also some concerns. For example, there is a worry that online help-seeking may delay access to formal help sources [10]. It is important to understand how these online resources, both formal and informal, are viewed by young people. Systematic reviews have been conducted in this area with a focus on how young people search for health-related information on the internet [15] and the effectiveness of online mental health services to improve help-seeking [14]; however, this review sought to understand the process and experiences of young people with regard to their online help-seeking experiences. The objectives of this narrative review [16] were to conduct a systematic analysis of the research on this topic and use the research to identify future opportunities for research and design that can improve the online help-seeking experiences of young people. The specific aims of this systematic review were as follows:

1. To examine the strategies employed by young people to search for help online for mental health difficulties.
2. To describe young people’s experiences of online help-seeking for mental health difficulties.
3. To identify the benefits of young people’s use of online mental health resources for help-seeking.
4. To identify the limitations of young people’s use of online mental health resources for help-seeking.

## Methods

### Search Overview

This review was conducted adhering to the Preferred Reporting Items for Systematic Reviews and Meta-Analyses guidelines and was registered on the PROSPERO database (PROSPERO registration number: CRD42017072487). On the basis of the aims of the study, inclusion and exclusion criteria were established to guide the subsequent search process.

### Search Strategy

The following 6 databases were searched from database inception (no limits were placed on the publication date as the evidence base in this area is very recent and to the author’s (CP, DCUCD, and DCHSE) knowledge, a systematic review of this nature has not been completed before): PsycINFO, Cumulative Index of Nursing and Allied Health Literature, PubMed, Cochrane Library, Association for Computing Machinery Digital Library, and Institute of Electrical and Electronics Engineers Xplore in August 2017. In addition, the reference lists of all the included studies were scanned for relevant papers. The search terms aimed to represent the primary concepts of *online help-seeking*, *mental health*, and *young people*. Keywords were generated for each of these concepts by examining the terminology used in review papers in the help-seeking literature, and the authors sought the guidance of a trained librarian in the formation of the search string. The search strings are included in Multimedia Appendix 1. In keeping with the emerging youth mental health paradigm as described in the *International Declaration on Youth Mental Health* [17], the studies were restricted to young people aged 25 years and younger. Only English-language studies were included. All studies identified in the database search were exported to a reference managing software (EndNote X8 for Mac, Clarivate Analytics), and duplicate records were deleted.

**Figure 2:**
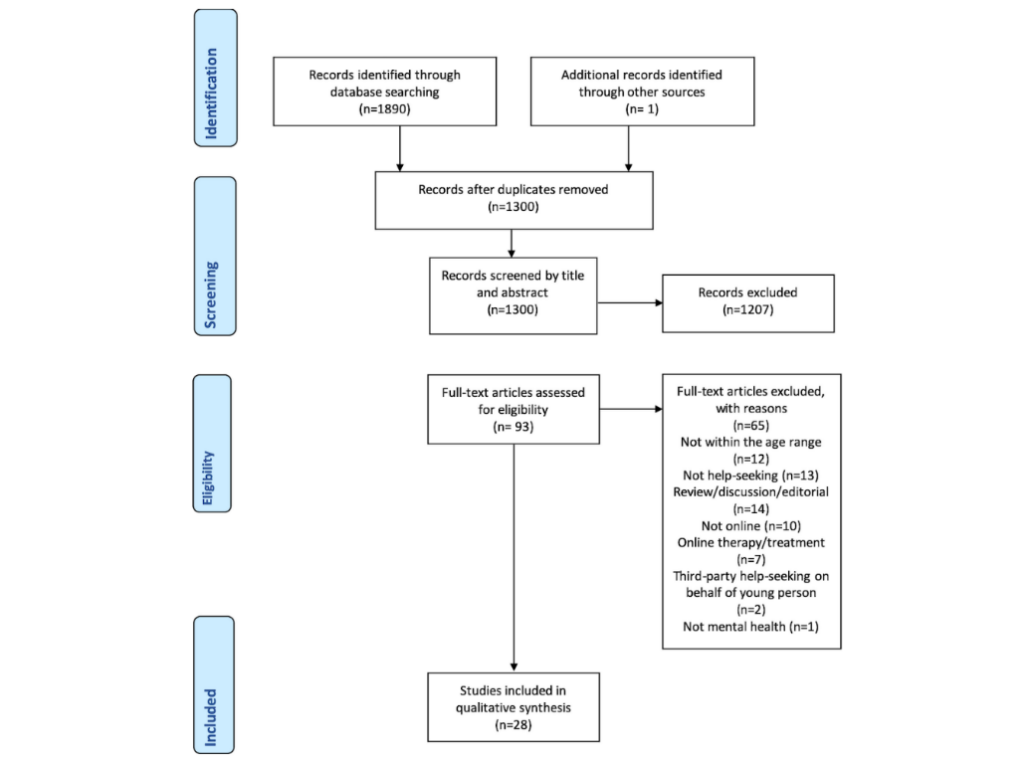
The Preferred Reporting Items for Systematic Reviews and Meta-Analyses (PRIMSA) flow diagram illustrating the screening process of papers.

The initial search identified 1890 published English-language abstracts (Figure 2). After removing the duplicates, 1300 papers remained. These papers were then reviewed by title and abstract to determine whether they met the inclusion criteria, resulting in 93 potentially relevant studies. At this stage, the full texts of these studies were obtained to confirm whether the inclusion and exclusion criteria listed below were met, resulting in 65 studies being excluded. The remaining 28 studies were included.

A random sample of 10% (130/1300, 10/93, 3/28) of the papers was re-examined at 3 stages (screening by title and abstract, screening by full text, and validity assessment) of the process by the other authors (DCUCD and DCHSE) of this paper. A few discrepancies were noted, and those were resolved by discussion and subsequent double checking to ensure consistency.

### Data Extraction

Each paper was read by CP and relevant details were extracted into a Microsoft Excel spreadsheet. There were a number of different protocol details that were examined and recorded for each paper. The coding scheme was used to help identify the components relevant to the study design and to address the research questions. The coding scheme included the year of publication, purpose of the study, country, number of participants, participants’ characteristics (eg, medical conditions and age), theoretical framework, research design, sampling, data collection methods, instruments (including reliability and validity), data analysis, help-seeking model employed, strategies found to be used by young people to seek help online, young people’s experiences of online help-seeking, facilitators, barriers, major findings, and study limitations.

### Analysis and Synthesis

Quality assessment of included studies was conducted using the Critical Appraisal Skills Program (CASP) checklists [18] as there are quantitative and qualitative versions available to allow appraisal across different study designs. The CASP checklists enable the assessment of trustworthiness, relevance, and results of published papers and are divided into 3 sections to assess internal validity, results, and relevance to practice. Quality criteria for surveys included sections on research question and design, sampling framework and participant understanding, instrument metrics, response rate, coding and analysis, and result presentation [19-21].

These sections are assessed by questions that can be answered with *yes*, *no*, or *can’t tell*. Each study received an overall rating of either *strong*, *moderate*, or *weak*, based on the number of questions scored as *yes*. Studies had to be scored as *yes* on majority of the questions to be rated *strong* overall (see Multimedia Appendix 2 tables for details, as criteria differed by study design).

The first author (CP) performed all of the quality assessments, with a 10% sample being given to the second author (DCUCD) to compare. Following the quality assessment stage, the inclusion of studies and extraction of key findings were finalized. Extracted data were entered into a table of study characteristics, including the quality assessment ratings for each study.

The decision was made to use narrative synthesis as it provides a broad overview of relevant information through a textual approach and is appropriate when it is expected that the studies will be heterogenous. Owing to the nature of the review questions, it was expected that the studies included would investigate help-seeking differently, make use of different research questions, and use different criteria to investigate help-seeking behaviors; thus, it would not be appropriate to make use of statistical synthesis techniques. The Guidance on the Conduct of Narrative Synthesis in Systematic Reviews [16] informed the data synthesis process to ensure that results and analysis were reported accurately. All the data extracted from the papers are presented narratively in text and summary tables.

### Eligibility Criteria

The inclusion and exclusion criteria are shown in Textbox 1.

**Inclusion and exclusion criteria.**

*Inclusion criteria*

- Young people not older than 25 years;
- Participants who present with psychological distress, self-selected general population samples, and those who have received a diagnosis from a health care practitioner;
- Studies designed to investigate and document the online help-seeking intentions and behaviors of young people;
- Studies that included an intervention or interventions that were designed to improve help-seeking attitudes or increase help-seeking intentions or help-seeking behaviors of young people;
- Web-based help-seeking interventions;
- Online mental health resources;
- Informal and formal help-seeking.

*Exclusion criteria*

- Studies focused on a third party seeking help for the young person;
- Not mental health related;
- Not a Web or mobile app–based;
- Unrelated technology, for example, computer games and their impact on mental health and social media and forums that are not specifically focused on mental health-related topics;
- Studies focused on online treatment methodologies and interventions, for example, computerized cognitive behavioral therapy or online counselling;
- A review paper.

## Results

### Study Characteristics

A total of 28 studies met the inclusion criteria (see Multimedia Appendix 3), of which the majority (n=16) were conducted in Australia. Others were conducted in the United Kingdom (n=4), Canada (n=4), the United States (n=2), Ireland (n=1), and the Netherlands (n=1). The studies were published between 2010 and 2017.

The research methodologies of the studies were varied. Only 4 of the 28 included studies mentioned which help-seeking model they employed to inform their study design [6,12,22,23]. These models were limited to Rickwood’s *help-seeking model* and *the theory of planned behavior*. Survey research was the most widely employed research design; overall 11 studies [24-33] made use of an online survey and 4 studies [34-37] administered the survey in person to collect data from participants. Survey questions included standardized measures such as the General Help-Seeking Questionnaire and mental well-being scales such as the Kessler Psychological Distress Scale, but questions regarding technology and internet use varied and no standardized scales seemed to be available. Areas explored in these sections of the surveys included the internet and social media use, use of online mental health resources, perceived usefulness of these resources, and preference regarding these resources. Other study designs included 1 randomized controlled trial [38], 1 feasibility study [39], 2 comparative studies [40,41], and 4 qualitative studies [9,42-44]. These studies aimed to assess the intention to seek help from different help sources, previous help-seeking behaviors from different help sources, level of psychological distress, and preferred modes of delivery of online mental health services.

The number of participants in each study ranged from 23 to 3946. Their ages ranged from 12 to 25 years. The majority of studies had gender-mixed samples; however, 3 studies looked specifically at the online help-seeking behavior of males [9,22,25]. In all, 9 studies recruited participants who were students at a university or high school [9,22,24,27,30,31,35,37,42], whereas 6 studies [25,26,28,29,32,33] used online recruitment strategies. Most studies (n=22) were conducted with samples not selected on the basis of mental health status. However, 5 studies focused on participants who had reported experiencing self-harm and suicidal ideation [24,28,36,43,45], and 1 study specifically recruited participants who had been diagnosed with psychosis and nonpsychotic mood disorders [46].

### Methodological Evaluation

Many of the studies achieved a strong or moderate rating on the checklists. However, the survey studies showed poor adherence or failed to report that they had indeed piloted the survey with a small number of young people before administering the surveys. Furthermore, randomized controlled trials showed small treatment effects, and there was poor evidence of rigorous data synthesis methods in the qualitative studies. See Multimedia Appendix 2.

### Limitations of the Studies

All of the studies included some notable limitations which indicate specific gaps in the literature and findings that may not be generalizable to other populations. Many of the studies reported having participants, where the majority were female [27-31,33,35,40,45]. Although some studies focused specifically on males, it must be considered that much of the evidence in this area is from a female perspective. Many of the studies were based in Australia, where there is a great deal of investment in youth mental health services compared with other countries in the world. In addition, many of the studies recruited from a university population only [24,27,30,31,43], who are not representative of young people in general. Finally, many of the studies were cross-sectional or retrospective studies [12,24,29,36,47]; these types of studies include recall bias and do not accurately account for actual future behaviors.

### How Young People Seek Help Online

In total, 6 studies found that young people made use of text-based queries using search engines to find mental health–related information rather than accessing a specific website [9,27,36,37,43,48] (see Multimedia Appendix 3). Information seeking about symptoms and forms of treatment are common goals when searching online [33]. The study by Birnbaum et al [47] showed that type of mental illness (mood disorder vs psychotic disorder) influenced what young people searched for. In this case, young people experiencing a mood disorder were more likely to search for how to stop symptoms, whereas those with a psychotic disorder preferred to understand why their symptoms had come about. When investigating the terms used by young people to search for mental health help, there was frequent use of *mental health*, *mental health problems*, *depression*, or *symptoms of...,* and *treatment of...* [30,37,43]. A common theme in the studies is the search for symptoms and treatment for the mental health concern the young person is currently facing [33]. The other types of content accessed that were identified by the studies include YouTube videos, factsheets, personal stories, and forums [33].

A total of 3 studies found that young people had used social media to locate mental health information [27,47,49]. The use of mental health or government websites varied from study to study. A study by Burns et al [48] found that less than 44.4% of the sample sourced information from mental health websites, similarly the study by Feng et al [27] found that only 26% of their sample had made use of information sites. Conversely, in a more recent study by Wetterlin et al [33], 82.9% of the sample indicated that they would be *somewhat likely* or *very likely* to use an information-based website. The study by Best et al [9] found that less of a quarter of their sample would make use of a government website.

Despite the lack of preference for formal mental health or government websites, a number of studies found that young people valued online services run by mental health professionals [9,28,47]. The study by Birnbaum et al [47] found that young people expressed an interest in obtaining help from mental health professionals through social media, whereas the study by Best et al [9] found that young people valued online services run by mental health professionals despite not wanting to use government websites. Haner and Pepler [40] found that the more distress the young person was experiencing the more likely they were to access the *Live Chat* option with a website providing mental health support to young people. Similarly, Frost et al [28] found that young people who self-harm would prefer an online service that allowed them to directly link with a mental health professional via instant messaging when in crisis.

Online communities and discussion forums also serve as a platform young people use to seek help. In the analysis of an online community, Greidanus and Everall [44] found that help-seekers would come to the forum to post messages seeking help for personal distress, looking for input from other users of the online community. The use of discussion boards or online support groups was reported by 11% of the sample in the study by Feng and Campbell [27] and 48.6% of the sample in a study by Frost et al [28].

### Motivating Factors for Young People to Seek Help Online

The studies identified by this review indicated that many young people were going online to look for a space where they could share their feelings without fear of judgement or labelling but at the same time, it was important that these spaces protected their privacy [30,33,43].

Many studies found that there is an association between high levels of psychological distress and engaging in help-seeking online [12,22,36,40,50]. Majority of this help-seeking tends to take place after 11 pm at night [22,48]. The study by Best et al [9] found that a preferred online service would be one run by professionals, available 24 hours a day. The need for services run and recommended by professionals is a recurrent theme throughout all of the studies [25,28,44]. A study by Wetterlin et al [33] found that 83.9% of participants reported that it was important to them to have human contact within an online mental health resource. This need for human contact also includes the need or desire to connect to peers online who can support the online help-seeking process; this is especially true of users of online support communities [30,33,37,41,43,44].

There seems to be a sentiment that there is more help available to young people online than offline, and that young people from minority groups and those with higher levels of psychological distress were more likely to disclose their current difficulties online rather than offline [32,35]. A study by Frost et al [28] indicated that this may be due to many young people finding online spaces to be less judgmental, and the support they received was nonstigmatizing. This is especially important because a number of studies found that those young people with increased levels of psychological distress were likely to access mental health content online but not seek help from offline sources [13,28,29,31,41].

### Young People’s Experiences of Online Help-Seeking

The findings regarding the perceived helpfulness of online resources were variable (see Table 1). Ellis et al [26] found that 81.9% of females in their sample reported that talking online had helped, and that they were either satisfied or very satisfied with the process. Similarly, 54.9% males had talked about their problems online, and 81.3% of men found that it had *helped* and were satisfied with the help they had received. The study by Feng and Campbell [27] found more mixed results with 59% of this sample indicating that the online resources they had made use of *didn’t make things better or worse* and 40% reported that they had *helped a little*. Ruppel and McKinley [31] investigated social anxiety and levels of social support in relation to the perceived usefulness of online resources; they found that participants with higher levels of social anxiety and also those with high levels of social support found online support groups to be very useful. The analysis of an online community by Greidanus and Everall [44] found that young people had experienced the online communities (a community message board) as understanding and affirming; this sentiment was especially strong for users who felt misunderstood offline. Overall, the comments indicated that users had found their engagement on the site to be a positive experience.

**Table 1:** Findings identified in studies: what are young people’s experiences of seeking help online?

Authors (year)	Findings related to young people's experiences online
Ellis et al (2013) [25]	Most females said that talking online *helped* (81.9%), and that they were *satisfied* or *very satisfied* with the online help they received. More than half of all male respondents reported that they had talked about their problems online (54.9%). Most said that talking online *helped* (81.3%), and that they were *satisfied* or *very satisfied* with the online help they received (82.9%).
Feng and Campbell (2011) [27]	In total, 59% of participants reported that online resources that they had used *didn’t make things better or worse*, 40% reported *they helped a little*, and only 1% of participants reported *they helped a lot*. Although there is a preference for text-based search engines and information sites, the current sample does not seem to find them to be efficacious.
Frost and Casey (2016) [29]	Over half of these online help-seekers perceived that they had more support available to them online than offline.
Frost et al (2016) [28]	Young people identifying the need for a nonjudgmental (n=68) and safe (n=14) environment and interactions. Many young people used the term nonjudgmental, whereas others indicated that they needed support in a way that was not stigmatizing, did not stereotype them, blame them, or label them as an attention seeker. Safety in online services for self-injury centered around the need for moderation, warnings about triggering content, and the risks of self-injury becoming competitive. Young people with a previous experience of online help-seeking were more likely to endorse the importance of reduced isolation and a supportive online culture.
Greidanus and Everall (2010) [44]	Most messages written by the trained volunteers took the form of an affirmation of some aspect of the help-seeker’s character. A strong sense of community was indicated in several of the threads when help-seekers stated they felt their experiences were understood and shared by other members. This sense appeared to be especially strong for those help-seekers who felt misunderstood by those in their *offline* lives. Most of the community members authored a number of threads themselves and posted in threads of other members, occasionally making reference to the content of other threads. Participant comments often indicated they found engagement on the site to be a positive experience and provided a place to express feelings, receive support, and obtain referrals.
Mars et al (2015) [36]	Almost a quarter of the sample had come across a site that discussed self-harm or suicide.
Ruppel and McKinley (2015) [31]	Participants with higher social support perceived websites and online support groups as more useful. The perceived usefulness of online support groups was highest among participants who had high levels of social anxiety and high levels of social support.
Wetterlin et al (2014) [33]	Most participants (87.7%) rated their privacy as a user as *very important*.

### Benefits of Online Help-Seeking

Online help-seeking offers a number of benefits to young people experiencing personal and emotional difficulties. A total of 14 studies identified benefits of online help-seeking (see Table 2). These benefits could be grouped into 8 overarching categories which have been included in Table 3.

A total of 8 studies found that the anonymity provided by the internet was an important facilitator to online help-seeking [9,25,30,35,37,44]. Similarly ease of access and the immediacy of the internet plays an important role in its attractiveness to young people [9,12,28-30,35,51]. The nonstigmatizing nature of internet help-seeking makes it an attractive option for marginalized groups as seen in the study by Haner et al [40]. These groups include migrants and members of the LGBT+ community who may be fearful of disclosing personal concerns to their informal networks [40]. Similarly, a study by Best et al [22] found that online help-seeking was not affected by socioeconomic status or educational attainment.

Young people are finding a sense of community online and are able connect with others who have similar experiences to their own [24,43,46]. They feel they are able to communicate with this community without fear of judgment and, more importantly, they can control their level of disclosure [9,28]. In all, 2 studies indicated that young people who had previously gone online to seek help for self-injury or suicide-related issues were significantly less likely to have disclosed to someone offline [24,28].

**Table 2:** Benefits of seeking help online by study.

Authors (year)	Findings related to benefits of online help-seeking
Bell et al (2018) [24]	Online help-seeking allows young people to communicate with others (social support but also reducing isolation). Information is readily available. Supportive sense of community and acceptance. Comfort and relief in realizing that they are not alone.
Best et al (2016) [9]	Anonymity Ease of access Immediacy Absence of judgement Can control level of disclosure
Best et al (2014) [22]	Some males may not disclose problems to others, but they are receiving some form of support through help-seeking practices online. Online help-seeking is not affected by Socio Economic Status or educational attainment. Online sources may be providing young males with an additional outlet to seek social support.
Birnbaum et al (2017) [47]	Opportunities for early intervention, as information found online can play an important role in the treatment-seeking decision-making process. Young people are fearful to talk to close others about their symptoms but are comfortable to use the internet for further understanding. Social media gives mental health clinicians the opportunity to engage and meaningfully interact with struggling youth at the earliest phases of illness potentially altering the trajectory to care. Online information seeking plays an important role in the initiation of help-seeking by influencing individual’s understanding of symptoms and their decision to seek professional help.
Bradford and Rickwood (2014) [35]	Anonymity Information that is easily accessible Finding others who have similar experiences.
Burns et al (2016) [13]	Reasons for preference of online resources included the anonymity of the internet, that information was easily accessible, and that there are often people in chat rooms who have been through the same thing. Boys were shown to have a stronger preference for online resources compared with face-to-face help relative to girls.
Burns et al (2010) [48]	Access online mental health resources in crisis outside of working hours (after 11 pm).
Collin et al (2011) [12]	Online help-seeking helps young people to be more willing to ask a professional for help. Upon having positive experience, help-seekers become advocates of help-seeking. Gateway services promote timeous help-seeking.
Ellis et al (2013) [25]	Preference instead for self-help and action-oriented strategies. The internet addresses their desire for anonymity and self-help.
Frost and Casey (2016) [29]	Online help-seekers indicated a greater intention to seek help for self-injurious behavior in the future. A significant difference in help-seeking intentions from professionals emerged, with online help-seekers indicating significantly higher intentions to seek professional help compared with individuals who did not seek help online. The internet may have an important role to play in mitigating help negation in young people who self-injure. Young people who sought help online in relation to self-injury indicated a significantly greater intention to seek help for self-injurious behavior in the future, even after controlling for age, gender, and psychological distress.
Frost et al (2016) [28]	Over half of the sample indicated a desire to use the internet as a first step but to later gain support offline. The internet may provide a way of accessing support that is perceived as remaining private and within the control of the young person. Perceived sense of community and belonging for young people who self-injure.
Greidanus and Everall (2010) [44]	It is clear these help-seekers, who reported not feeling comfortable seeking help from professional *offline* services, were able to use internet-based communication to create a community where they found support and offered support to their peers. Children and adolescents who use alternative communication technologies find internet-based communications meaningful and personally relevant. Help-seekers identified anonymity, accessibility, and access to peers who understand their experiences as important aspects of online help. Help-seekers reported finding it easier to disclose some experiences online than offline.
Horgan and Sweeney (2010) [30]	Anonymity, privacy, and conﬁdentiality. Accessibility, speed, and cost. Believed that they would not be judged and believed it would be a good place to get initial information. Easier to express themselves. Ability to communicate with others in similar situations to ﬁnd out how they are coping. Young people indicated they are less likely to lie online. Young people are reluctant to access mainstream mental health services because of fear of judgement and because of the stigma that still exists in relation to mental health problems.
Mar et al (2014) [43]	Participants recounted using the internet to find others coping with similar problems, research their symptoms and prescribed medications, or understand their diagnosis. Participants also emphasized that knowing there is a community of others helps them to recognize that they are not alone with their problems. Participants sought a variety of e-mental health features, especially for engaging in active coping, such as journaling. Online services may afford them a level of privacy. E-mental health services may lessen the burden on providers or provide resources for patients waiting to access care. E-mental health services may help to treat those with mild symptoms or those who do not wish to seek professional support. Online services may also help direct those in need to the traditional health care system.

**Table 3:** Key benefit themes and number of studies in which each theme is addressed.

Serial no	Benefit	Number of studies	Studies
1	Anonymity and privacy	8	Best et al (2016) [9]; Bradford and Rickwood (2014) [35]; Burns et al (2016) [13]; Ellis et al (2013) [25]; Frost et al (2016) [28]; Greidanus and Everall (2010) [44]; Horgan and Sweeney (2010) [30]; Mar et al (2014) [43]
2	Ease of access and immediacy	7	Bell et al (2018) [24]; Best et al (2016) [9]; Bradford and Rickwood (2014) [35]; Burns et al (2016) [13]; Burns et al (2010) [48]; Greidanus and Everall (2010) [44]; Horgan and Sweeney (2010) [30]
3	Connecting with others with similar experiences	7	Bell et al (2018) [24]; Bradford and Rickwood (2014) [35]; Burns et al (2016) [13]; Frost et al (2016) [28]; Greidanus and Everall (2010) [44]; Horgan and Sweeney (2010); Mar et al (2014) [43]
4	Acts as a gateway to further help-seeking	5	Birnbaum et al (2017) [47]; Collin et al (2011) [12]; Frost and Casey (2016) [29]; Frost et al (2016) [28]; Mar et al (2014) [43]
5	Increased perceived control of help-seeking journey	3	Best et al (2016) [9]; Frost et al (2016) [28]; Mar et al (2014) [43]
6	Meets the needs of those with a preference for self-reliance	2	Ellis et al (2013) [25]; Mar et al (2014) [43]
7	Early access	2	Birnbaum et al (2017) [47]; Frost et al (2016) [28]
8	Inclusiveness of different Social Economic Status/cultures/genders	1	Best et al (2016) [22]

Online help-seeking seems to act as a gateway behavior to further help-seeking. It enables young people to access information about their mental health difficulties and, therefore, decide whether there is a need to seek professional help [9,12,28,46,49]. The internet provides alternative routes to access mental health professionals; for instance, Birnbaum et al [47] found that young people would be willing to access opportunities to connect with clinicians over social media. Collin et al [12] investigated the role of an online youth mental health website, ReachOut.com, in promoting young people’s help-seeking behavior. Users of the website (43.3% of those surveyed) indicated that using the website had helped them to acquire the skills and confidence to seek help if they needed it. Online resources could have a role to play in early intervention as the information found online could help early identification of concerning symptoms but also assist in reaching out to mental health professionals.

The internet also provides access to the information and tools that may assist those young people who have a preference for self-reliance or for informal sources of help [25,44]. In the study by Mar et al [43] participants indicated that they used the internet to search for active coping strategies such as journaling to assist them to cope with their current difficulties.

### Limitations to Online Help-Seeking

Limitations to online help-seeking were discussed by 14 studies with 6 common categories of limitations found across all the studies (see Tables 4 and 5).

Young people are motivated to look for help online; however, their ability to access reliable, helpful information is influenced by their lack of mental health literacy and a lack of knowledge of which resources to search for [27,31,43,49]. In the context of help-seeking, mental health literacy can be understood as knowledge and understanding of mental health problems which aid their recognition and management [52]. Formal online services are limited and would need to be familiar to the help-seeker to be accessed [9]. A key concern raised by young people across 3 studies is that they were uncertain whether certain sources are reliable or not and lack an understanding of the indicators of quality [9,23,30]. It appears that young people attribute quality based on superficial characteristics such as rank on Google search results and design and layout of websites [9].

Treatment avoidance is a real risk associated with online help-seeking. Content exists online that can be stigmatizing, triggering, or that may reinforce harmful behaviors and thoughts [24,28]. Certain communities may also perpetuate the stigma surrounding mental health and psychiatric treatment options, which may contribute to a reluctance to seek help from offline, professional services [13,47]. The usual protective measures are not present in unmoderated communities, and it is concerning that risky content may not be removed [24]. This risk is exacerbated as young people may incorrectly attribute certain sources as *helpful* when in fact, they are dangerous [9,30,36,47]. Rickwood et al [41] expand on this by emphasizing that the self-reliance afforded by online help-seeking may have limited young people’s access to the appropriate help source at the appropriate time because of limits of their own mental health literacy.

Finally, young people are concerned about the implications of making use of online help-seeking. These include fears over protection of privacy, that it may be too impersonal, and that the help found there would be unreliable and untrustworthy [30,39,43]. Mar et al [43] found that young people’s concerns regarding their privacy centered around fears that family and friends would somehow find out about their mental health concern. A concern many of them also have about offline help-seeking.

**Table 4:** Themes identified in studies: limitations of seeking help online.

No.	Limitation	Number of studies	Studies
1	Uncertainty about trustworthiness of resources	5	Best et al (2016) [9]; Kauer et al (2017) [39]; Horgan and Sweeney (2010) [30];
2	Lack of mental health literacy	5	Bell et al (2018) [24]; Best et al (2016) [9]; Feng and Campbell (2011) [27]; Mar et al (2014) [43]; Ruppel and McKinley (2015) [31]
3	Reinforcing treatment avoidance	4	Birnbaum et al (2017) [47]; Mars et al (2015) [36]; Rickwood et al (2015) [41]
4	Concerns about privacy and confidentiality	3	Best et al (2016) [9]; Horgan and Sweeney (2010) [30]; Mar et al (2014) [43]
5	Triggering negative behavior	2	Bell et al (2018) [24]; Mars et al (2015) [36]
6	Difficulty in providing an emergency response	1	Mar et al (2014) [43]

**Table 5:** Limitations of seeking help online by study.

Author (year)	Findings related to limitations of online help-seeking
Bell et al (2018) [24]	The risk of triggering or reinforcing suicidal thoughts or behaviors. Unmoderated communities are risky as the fail-safe to remove risky content is not there.
Best et al (2016) [9]	Lack of understanding of indicators of quality. Lack of control of personal information once it is online. Lack of confidentiality when disclosing within your own social network. Lack of help-seekers’ health literacy. Formal online resources are limited and need to be known to be accessed.
Birnbaum et al (2017) [47]	The online environment can be misleading and stigmatizing that reinforces pre-existing misconceptions about mental health and psychiatric treatment options, which may contribute to treatment avoidance.
Burns et al (2016) [13]	There is still an overall orientation to not seek help, and barriers remain to all forms of help. This has concerning implications as it suggests that simply providing help through different means will not increase the likelihood that young people facing these barriers will actually use these new avenues of help.
Collin et al (2011) [12]	Despite overall increased mental health literacy and intentions to seek help, ReachOut.com visitors remain reluctant to seek help from traditional and face-to-face sources.
Feng and Campbell (2011) [27]	Young people are unaware of where to search for mental health concerns.
Frost et al (2016) [28]	It is unclear whether online help-seeking was acting to replace offline help-seeking for these young people or whether the internet facilitates help-seeking in young people who otherwise would not disclose their self-injury to anyone. Similarly, it is unclear whether the failure of these young people to seek help offline may reflect a lack of linking to offline support in current forms of online support for self-injury. Young people in the current sample went beyond discussion of the positive aspects of online communities and online culture, expressing concerns about triggering content, unmoderated discussions, and the *glorification* of self-injury.
Haner and Pepler (2016) [40]	The possibility exists that the online counsellors can misinterpret neutral or positive typed communication with the presence of a vocal cue to suggest warmth of tone.
Horgan and Sweeney (2010) [30]	A number of participants also reported that they believed it would be unreliable (15.1%), untrustworthy (5%), it lacks privacy (2.5%), is too impersonal (7.5%), and that insufﬁcient support would be found (3.9%). A number of participants were concerned with the reliability of the information, highlighting that young people may be experiencing difﬁculty in determining the quality of information online.
Kauer et al (2017) [39]	Lack of trust in the accuracy of the information available on the internet was also a general concern for both the Link and comparison arms.
Mar et al (2014) [43]	Two participants spoke of the importance of advertising the existence of online support offline, explaining that they felt it was not intuitive to look for help online. Participants’ concerns over privacy generally linked back to the stigma of having friends or family find out about their mental health concern. Providers of e-mental health services for youth must appropriately address high suicide risk while maintaining a youth’s privacy, which may need to be breached in emergency circumstances.
Mars et al (2015) [36]	Young people have difﬁculties in classifying sites as either *helpful* or *harmful*, as some offer concurrent suicide-promoting and help-promoting content.
Rickwood et al (2015) [41]	Greater self-reliance online, with a slightly stronger peer influence, may be cause for concern, as young people and their friends may not be the best guides to appropriate mental health care.
Ruppel and McKinley (2015) [31]	Limited mental health literacy and limited knowledge about which resources are available.

## Discussion

### Principal Findings

This review aimed to extend understanding of how young people use online resources to seek help for their mental health concerns. A total of 28 studies were identified. Only 4 studies explicitly identified a theoretical framework of help-seeking that guided the study design. Moving forward, the development of such theoretical frameworks represents a key challenge. Results suggest that the internet serves 3 functions to help-seekers: (1) as a gateway to further information and knowledge acquisition around their symptomology; (2) as a way to connect with others, professional or peer, around the topic of their mental health difficulties; and (3) as an alternative option to offline help-seeking for those who are most at risk. A text-based query via an internet search engine was the most commonly identified help-seeking approach. But social media, government or charity websites, live chat, instant messaging, and online communities and discussion forums are also used. The perceived benefits of online help-seeking include anonymity and privacy, ease of access, inclusivity, and the ability to connect with others and share experiences. Online help-seeking may also increase young peoples’ sense of control over their help-seeking journey; meet the needs of those with a preference for self-reliance; or act as a gateway to further help-seeking. In contrast, significant limitations were also identified. A lack of mental health literacy can act as a barrier to effective help-seeking, as can concerns about privacy and confidentiality, and uncertainty about the trustworthiness of online resources. There is a concern that online help-seeking can reinforce treatment avoidance or trigger negative behavior.

### Theoretical Frameworks in Online Help-Seeking

This review highlights the limited use of theoretical frameworks to help conceptualize online help-seeking and guide the development of improved resources. The full development of such a theory is beyond the scope of this paper. However, we consider 2 potential starting points for such a theory: Rickwood et al’s *help-seeking model* [3] and *self-determination theory* (SDT) [53]. In each case, we use the existing theory as a lens through which to analyze the benefits and limitations of online help-seeking identified in this review and how they can either support or frustrate the help-seeking process.

#### The Help-Seeking Model

Existing theories of help-seeking provide a valuable starting point for the development of online help-seeking theories. The *help-seeking model* of Rickwood et al [3] provides a stage-based model to understand traditional help-seeking behaviors. It was applied by 2 studies in this review. Best et al [9] have proposed a pathways-based extension of this theory through their *pathways to online help-seeking model*, which seeks to predict people’s help-seeking decisions on the basis of their mental health literacy and perception of stigma.

Table 6 demonstrates another approach to applying the *help-seeking model*. It outlines 1 way in which the benefits and limitations identified in this review can be mapped to stages of the *help-seeking model*. Through such a mapping, we can begin to identify and think about important issues that impact different stages of an online help-seeking process. For example, early access and the potential of online services to act as a gateway to further help-seeking may offer significant benefit at the awareness stage of a help-seeking process. This is offset by the lack of mental health literacy many young people will have at this stage of the process. Similarly, a lack of literacy is also likely to impact the expression stage. However, while young people may struggle to recognize or express their symptoms using formal clinical language, they might benefit from reading the stories of other young people, whose experiences they might relate to, potentially helping them to understand their own symptoms in a more accessible manner, and thus enabling expression.

As shown in Table 6, this approach can also be applied at the availability and willingness stages. In each case the approach provides a structured way to think about benefits we might maximize, while also highlighting limitations that need to be addressed. Such an analysis can guide the design of more effective online help-seeking services. It is also important to note the we do not see the mapping presented here as exclusive. We recognize that other mappings are possible. Our intention is to demonstrate how consideration of distinct benefits and limitations at each stage of a help-seeking model can shed light on key challenges and opportunities in the design of online help-seeking services.

**Table 6:** A mapping of the benefits and limitations to online help-seeking based on the stages of *help-seeking model* by Rickwood et al [3].

Stage	Awareness	Expression	Availability	Willingness
Process	Becoming aware of symptoms, appraising the assistance required	Expressing the symptoms experienced and that they are in need of help or support	Identify sources of help that are available and accessible	Willingness of the help-seeker to disclose difficulties to the selected, available source
Benefit/support	Early access Acts as a gateway to further help-seeking	Connecting with others with similar experiences	Ease of access and immediacy Inclusiveness Meets the needs of those with a preference for self-reliance	Anonymity and privacy Control of help-seeking journey Connecting with others with similar experiences
Limitation/frustration	Lack of mental health literacy	Lack of mental health literacy	Lack of immediate, crisis support	Concerns about privacy and confidentiality Treatment avoidance Triggering negative behavior

#### Self-Determination Theory

SDT is a theory of motivation that has been applied across many settings in education and health care to understand and predict psychological well-being [53,54]. In recent years, it has also been applied in the design of digital technologies that can support mental health and well-being [55,56]. Until now, it has not been applied to online help-seeking.

SDT consists of a number of mini-theories; one of which is *cognitive evaluation theory* [53,54]. This theory proposes that there are 3 primary psychological needs for well-being and motivation: autonomy (to experience choice in line with one’s own interests and values); competence (effectively interact with one’s environment and express one’s abilities); and relatedness (sense of belonging) [57]. SDT postulates that these basic 3 needs are essential for understanding how and why humans pursue certain goals. It asserts that the natural human trajectory is toward vitality, integration, and health [58] and argues that environments can support or frustrate these needs and thus influence well-being and motivation.

Here, we consider how SDT can be applied to conceptualize motivation in online help-seeking. In Table 7, the benefits and limitations of online help-seeking identified in this review are clustered in terms of their impact on autonomy, competence, and relatedness as consistent with SDT. In online help-seeking, the goal might vary from person to person; however, those specific goals could be understood within the broad themes of achieving growth and well-being. One could, therefore, argue that online searches and resources that are designed to support these basic psychological needs will be better able to support young people when they engage in help-seeking.

The evidence from this review suggests that there were mixed responses with regard to young people’s satisfaction with their experiences when looking for help online. This mixed response could be attributed to online resources not fully meeting the needs for autonomy, competence, and relatedness, or these needs only being partially met. An example of this could be the use of a text-based search engine, which facilitates the need for autonomy, but leads to an abundance and variety of search results that could overwhelm the young person, frustrating their need for competence. The mapping also shows how a lack of mental health literacy can be thought of as an issue of competence. Addressing this competence may increase overall motivation for help-seeking. Similarly, the decision to avoid treatment may be a negatively focused expression of autonomy. In such a conceptualization, we might predict that systems which support alternative forms of autonomy, for example, by making control of the journey more explicit, will reduce the likelihood of treatment avoidance.

The importance of connecting with others online, whether professionals or peers, is emphasized by participants in many of the reviewed studies, indicating the importance for a human element in both formal and informal online help sources. Previous research has also shown that knowing someone who has sought help for a mental health difficulty has a positive effect on one’s attitude toward help-seeking [3,53,54]. Viewed through the lens of SDT, engaging with people online or with content that shares the stories of other’s help-seeking journeys helps to provide relatedness and improve mental health literacy. As such the internet may play an important bridging role between different stages of a help-seeking process, first facilitating informal contact, but also increasing motivation toward formal help-seeking.

**Table 7:** A clustering of the benefits and limitations to online help-seeking on the basis of the primary psychological needs identified in self-determination theory.

Benefits and Limitations	Autonomy	Competence	Relatedness
Benefit/support	Anonymity and privacy Ease of access and immediacy Control of help-seeking journey Meets the needs of those with a preference for self-reliance	Acts as a gateway to further help-seeking Early access	Connecting with others with similar experiences Inclusiveness
Limitation/frustration	Concerns about privacy and confidentiality Treatment avoidance	Lack of mental health literacy	Lack of immediate, crisis support Triggering negative behavior

### Tensions and Opportunities in Online Help-Seeking

Evidence suggests that the internet has the potential to serve as an inclusive gateway that assists all young people in accessing help, especially those from minority groups and groups who experience a great deal of stigma. It provides immediacy of access and allows people to connect with others, while also preserving the option to remain anonymous and control how much information they reveal, thereby supporting relatedness and also meeting the need for autonomy. However, many young people may feel forced to limit what they reveal or how they search due to concerns around privacy and confidentiality, now limiting their autonomy. This highlights a tension between the potential benefit of human contact versus the need for confidentiality. Relatedly, there is a tension between the preference of some young people for self-reliance versus the benefits of disclosure to formal sources of help.

Rickwood et al’s model emphasizes the importance of the social transactions implicit in traditional offline help-seeking [3]. However, online help-seeking changes the nature of and need for social transactions with others. This review has found that while some online help-seekers prefer online mental health resources that offer the opportunity to connect with others, others prefer to navigate the process on their own and rely on self-help strategies, removing the need for a social transaction. The different types of content on the internet mean that the nature of social transactions has also changed: a help-seeker can now read content regarding another young person’s personal experiences of mental health difficulties without ever directly engaging with the original writer of the content. The writer of the content can still have a profound effect on the help-seeker, not only improving their mental health literacy but also providing an example of someone who has also sought help for a mental health difficulty. In this way, help-seekers become the consumers as well as the creators of help-seeking content online.

Online help-seeking can facilitate young people’s autonomy by allowing them to control their help-seeking journey. However, as noted, young people have differing preferences for which online resources they access and which resources they find useful. These preferences are not just different from person to person. They may also differ for any given person, depending on their immediate circumstances. A key challenge for future research lies in providing tailored and appropriate online resources for different preferences and groups that meet all 3 psychological needs. Addressing all 3 needs in an online resource currently appears to be lacking. For example, young people are accessing resources where they can read and share personal stories, which meets their need for relatedness but it is unlikely that they would meet their needs for competence (is this information trustworthy?) and autonomy (is my privacy ensured?) through these resources.

Our analysis suggests that young peoples’ online help-seeking may trigger key tensions between supporting and frustrating the 3 basic psychological needs outlined in SDT; simultaneously, the internet and online resources have the opportunity and capability to address these needs through careful and considered design. Managing these tensions will be important if we are to realize the full potential of systems that support online help-seeking.

### Implications for Practice

As the internet becomes increasingly a part of everyday life and is seen as an accessible tool for information, it is important to have an understanding of how young people use the internet to meet their mental health needs. A plethora of online resources exist, both good and bad, and we only have a limited understanding of the patterns and characteristics of young people’s mental health–related internet use. Online help-seeking provides an added space for young people to access help sources; however, it is an addition to offline help-seeking and not a replacement. There remains a great need to educate young people to facilitate competent and appropriate help-seeking behavior, both online and offline. Online sources need to be designed with young people’s needs in mind, specifically making services available after-hours and providing access to trained professionals and peers. To increase reach, offline service providers need to consider online/digital strategies to offer a continuum of services to address the mental health needs of young people. Similarly, those developing online resources for young people need to do so in collaboration with professionals and young people.

### Limitations of This Review

This review has several limitations. Although a number of databases have been included, the choice of keywords may have resulted in missing relevant research. Owing to the exploratory nature of this review, the decision was made to include a wide range of study designs, and the review will ultimately be limited by the design of the studies included. Although strategies to limit bias were included through consultation with the second and third reviewer, the possibility of subjectivity in analyzing the findings is acknowledged. Additionally, the measures used in the studies were varied and samples were heterogenous, making it a challenge to compare outcomes across studies. It is also evident from the studies included in this review that further investigation is needed into the online help-seeking behaviors of young people from populations other than those included in these studies. These data are representative of a mostly female, university student sample, often living in Australia. These findings may not translate well onto other populations such as those young people living in Europe, Asia, or Africa, young men and those young people who have not accessed tertiary education.

### Conclusions

A key concern for researchers in this area should be the development of a model or framework in which to explain the motivations and benefits of online help-seeking and how it fits in with the overall help-seeking process for young people. The conceptualization of such a model would contribute to the cross-validation of findings and provide the ability to determine patterns. This review has considered *the help-seeking model* and SDT as valuable starting points for such a theory. It would allow research questions to be framed within the SDT constructs and theories, allowing for comparison and validation.

## Appendix

Multimedia Appendix 1 Search Strings.

Multimedia Appendix 2 Quality Checklists.

Multimedia Appendix 3 Tables 1 and 2.

## Abbreviations

CASP: Critical Appraisal Skills Program
SDT: self-determination theory
