# Supplementary material for: Young People’s Online Help-Seeking and Mental Health Difficulties: Systematic Narrative Review
Source: J Med Internet Res. 2019 Nov 19;21(11):e13873. doi: 10.2196/13873 (PMC6891826; doi:10.2196/13873)
Supplement: Multimedia Appendix 1 [file jmir_v21i11e13873_app1.pdf]

Multimedia Appendix 1: Search String

PsychINFO

1. "Help seek*" OR "Seek* help" OR "Seek* treat*"

AND

2. "Web-base*" OR Online OR e-health

AND

3. Mental OR Depress* OR Anxiety

MySearch:

su(help seek* OR seek* help OR help seeking behaviour OR Health care seeking behaviour OR self-referral OR self help techniques) AND su(web-base* OR online OR e-health OR Computer Applications OR mobile devices OR Internet OR Information Technology) AND su(mental health OR Mental Disorder OR Mental OR Depress* OR Anxiety)

PUBmed

1. Diagnostic Self Evaluation* OR Self Assessment OR Information Seeking Behaviour OR Help Seek* OR Seek help* OR Self refer*

AND

2. Internet OR Cellular Phone OR Medical Informatics OR Software OR Web Base* OR e-mental health OR Information Technology OR Website OR Online OR Computer Application

AND

3. Mental Health OR Mental Disorders OR Mental OR Depress* OR Anxiety

((("Diagnostic Self Evaluation*" OR "Self Assessment" OR "Information Seeking Behaviour" OR "Help Seek*" OR Seek help* OR "Self refer*")) AND (Internet OR "Cellular Phone" OR "Medical Informatics" OR Software OR "Web Base*" OR "e-mental health" OR "Information Technology" OR Website OR Online OR "Computer Application")) AND ("Mental Health" OR "Mental Disorders" OR Mental OR Depress* OR Anxiety)

Cochrane Library

“Diagnostic Self Evaluation*" OR "Self Assessment" OR "Information Seeking Behaviour" OR "Help Seek*" OR Seek help* OR "Self refer*" AND Internet "Cellular Phone" or "Medical Informatics" or Software or "Web Base*" or "e-mental health" or "Information Technology" or Website or Online or "Computer Application" and "Mental Health" OR "Mental Disorders" OR Mental OR Depress* OR Anxiety

SCOPUS

(TITLE-ABS-KEY ("help-seek*" OR "seek* help" OR "seek* treat*" OR "help seeking behaviour" OR "Health care seeking behaviour" OR "self-referral" OR "self help techniques") AND TITLE-ABS-KEY ("web base*" OR online OR e-health OR "Computer applications" OR "mobile devices" OR "Internet" OR "Information technology") AND TITLE-ABS-KEY ("mental health" OR "mental disorder" OR mental OR depress* OR anxiety))
