# Supplementary material for: Young People’s Online Help-Seeking and Mental Health Difficulties: Systematic Narrative Review
Source: J Med Internet Res. 2019 Nov 19;21(11):e13873. doi: 10.2196/13873 (PMC6891826; doi:10.2196/13873)
Supplement: Multimedia Appendix 2 [file jmir_v21i11e13873_app2.pdf]

Multimedia Appendix 2: Quality Assessment Tables

Quality Assessment for qualitative studies

1. Was there a clear statement of the aims of the research?
2. Is a qualitative methodology appropriate?
3. Was the research design appropriate to address the aims of the research?
4. Was the recruitment strategy appropriate to the aims of the research?
5. Was the data collected in a way that addressed the research issue?
6. Has the relationship between researcher and participants been adequately considered?
7. Have ethical issues been taken into consideration?
8. Was the data analysis sufficiently rigorous?
9. Is there a clear statement of findings?

Authors (year)	1	2	3	4	5	6	7	8	9	Rating
Best et al (2016).	Yes	Yes	Yes	Yes	Yes	Yes	Yes	Yes	Yes	Strong
Greidanus & Everall (2010).	No	Yes	Yes	Yes	Yes	Yes	Yes	Yes	Yes	Strong
Havas et . (2011).	Yes	Yes	Yes	Yes	Yes	Can't Tell	Yes	Can't Tell	Yes	Moderate
Mar et al(2014).	Yes	Yes	Yes	Yes	Yes	Yes	Yes	Can't Tell	Yes	Strong

Table 7. Quality Assessment for Survey/Questionnaire Studies

1. Did study address a clearly focused issue?
2. Was a questionnaire the most appropriate research design for this question?
3. Was the sample recruited in an acceptable way?
4.What claims for reliability and validity have been made, and are these justified?
5. Were open-ended (qualitative) and closed-ended questions used appropriately?
6. Was a pilot version administered to participants representative of those in the sampling frame, and the instrument modified accordingly
7. What was the response rate and have non-responders been accounted for?
8. Was the analysis appropriate (eg statistical analysis for quantitative answers, qualitative analysis for open/ended questions) and the correct technique/s used?
9. Have all relevant results (‘significant ‘and ‘non-significant’)?
10. Do you believe the results?
11. Can results be more widely applied?
12. Do the results of this study fit with other available evidence

Authors (year)	1.	2.	3.	4.	5.	6.	7.	8.	9.	10.	11.	12/	Overall quality rating
Bell et (2018).	Yes	Yes	Yes	Yes	Yes	Yes	Yes	Yes	Yes	Yes	Yes	Yes	Strong
Best, Manktelow, & Taylor (2016)	Yes	Yes	Yes	Yes	Yes	Not sure	Yes	Yes	Yes	Yes	Yes	Yes	Strong
Birnbaum et al (2017),	Yes	Yes	Yes	Yes	Yes	Not sure	No	Yes	Yes	Yes	Yes	Yes	Moderate

Bradford.& Rickwood, (2014),	Yes	Yes	Yes	Yes	Yes	Yes	Yes	Yes	Yes	Yes	Yes	Yes	Strong
Burns et al (2016).	Yes	Yes	Yes	Yes	Yes	Yes	No	Yes	Yes	Yes	Yes	Yes	Strong
Burns et al (2010).	Yes	Yes	Yes	No	Yes	Not sure	No	Yes	Yes	Yes	Yes	Yes	Moderate
Ellis et al. (2013).	Yes	Yes	Yes	No	Yes	Not sure	No	Yes	Yes	Yes	Yes	Yes	Moderate
Ellis et al(2012).	Yes	Yes	Yes	No	Yes	Not sure	Yes	Yes	Yes	Yes	Yes	Yes	Moderate
Feng, & Campbell (2011).	Yes	Yes	Yes	Yes	Yes	Not sure	Yes	Yes	Yes	Yes	Yes	Yes	Strong
Frost & Casey (2016)	Yes	Yes	Yes	Yes	Yes	Not sure	Yes	Yes	Yes	Yes	Yes	Yes	Strong
Horgan & Sweeney (2010).	Yes	Yes	Yes	Yes	Yes	Not sure	Yes	Yes	Yes	Yes	Yes	Yes	Strong
Mars et al (2015).	Yes	Yes	Yes	Yes	Yes	Not sure	Yes	Yes	Yes	Yes	Yes	Yes	Strong
Mok, Jorm & Pirkis (2016)	Yes	Yes	Yes	Yes	yes	Not sure	No	Yes	Yes	Yes	Yes	Yes	Moderate
O'Dea & Campbell (2011).	Yes	Yes	Yes	No	Not sure	Not sure	Yes	Not sure	Not sure	Yes	Yes	Yes	Weak
Ruppel & McKinley (2015).	Yes	Yes	Yes	Yes	Yes	Not sure	No	Yes	Yes	Yes	Yes	Yes	Moderate
Spears et al (2015).	Yes	Yes	Yes	Yes	Yes	Yes	Yes	Yes	Yes	Yes	Yes	Yes	Strong

Quality Assessment for Cohort Studies

1. Did study address a clearly focused issue?
2. Cohort/ sample recruited in an acceptable way?
3. Exposure accurately measured to minimise bias?
4. Outcome accurately measured to minimise bias?
5. Have authors identified important confounding factors?
6. Have authors accounted for confounding factors in design and/or analysis?
7. Do you believe the results?
8. Can results be more widely applied?
9. Do the results of this study fit with other available evidence?

**Authors (year)**	**1**	**2**	**3**	**4**	**5**	**6**	**7**	**8**	**9**	**Overall quality rating**
Barker et al (2017).	Yes	Not sure	Yes	Yes	No	No	Yes	Yes	Yes	Moderate
Haner & Pepler, D. (2016).	Yes	Yes	Yes	Yes	Yes	Not sure	Yes	Yes	Yes	Strong
Rickwood, Mazzer, & Telford (2015).	Yes	Yes	Yes	Yes	Yes	Yes	Yes	Yes	Yes	Strong

Quality Assessment Table for RCT Studies

1. Did study address a clearly focused issue?

2. Was the assignment of participants to conditions randomised?
3. Were all of participants who entered the trial properly accounted for at its conclusion?
4.Were participants, health workers and study personal ‘blind’ to treatment?
5. Were the groups similar at the start of the trial?
6. Aside from the experimental intervention, were the groups treated equally?
7. How large was the treatment effect?
8. Will the results help locally?
9. Can the results be applied to the local population?
10. Were all clinically important outcomes considered?
11. Are the benefits worth the harms and costs?

**Authors (year)**	**1**	**2**	**3**	**4**	**5**	**6**	**7**	**8**	**9**	**10**	**11**	**Overall quality rating**
Kauer et al (2017)	Yes	Yes	Yes	Yes	Yes	Yes	Not large	Yes	Yes	Yes	Yes	Strong
Sanci, Kauer, & Buhagiar (2017)	Yes	Yes	Yes	Not Sure	Yes	Yes	Not large	Yes	Yes	Yes	Yes	Strong
