# Supplementary material for: Young People’s Online Help-Seeking and Mental Health Difficulties: Systematic Narrative Review
Source: J Med Internet Res. 2019 Nov 19;21(11):e13873. doi: 10.2196/13873 (PMC6891826; doi:10.2196/13873)
Supplement: Multimedia Appendix 3 [file jmir_v21i11e13873_app3.pdf]

Table 1. Overview of studies included in final review.

Authors (year)	Location	Research methodology	Participants	Key findings
Barker et al (2017) [50]	Australia	An online screen consisting of the Alcohol Use Disorders Identification Test (AUDIT), Alcohol, Smoking, and Substance Involvement Screening Test (ASSIST), ASSIST], Kessler Psychological Distress Scale(K10), and Australian Treatment Outcomes Profile (ATOP)	Young people aged 15-24 years; (females =295, males =245)	• Young people access online tools with many being first time help seekers.<br>• An online tool, such as a screen, is an effective way to identify and engage help seekers.<br>• The online screen was effective in raising awareness and insight but also provided suitable pathways to support online and offline.

Bell et a (2018) [24]	United Kingdo m	Online survey	University students aged 18-24 years	• Studies revealed significantly higher levels of suicide risk and social anxiety among suicide-related internet users when compared with nonsuicide-related users.<br>• Significantly more nonsuicide-related users reported that they can talk about problems with their family than suicide-related users.<br>• Suicide-related internet users are a high-risk group who turn to the internet as their suicidal feelings increase.<br>• The question of what is harmful or helpful depends on the perceiver.<br>• It is important that sites are moderated by administrators who operate rules against unsupportive communication and remove disruptive or overly pessimistic content.<br>• A degree of caution is needed when assuming that sites dedicated to information about suicide methods and their effectiveness will always have a harmful or negative impact on suicidal internet users.
Best et al (2014) [22]	Norther n Ireland	48-item questionnair e: mental well-being; social networking	High school students, males aged 14-	• Searching online for health information was not associated with decreases in mental well-being among respondents.<br>• Very few respondents stated they would be likely to access mental

		and internet use; self-efficacy; online help seeking	15 years, (N=527)	health information through government health website.<br>• Knowing that the information came from a *trusted source* and the constant presence of a *trained professional* were the most importance features of online mental health services.<br>• Socioeconomic background and educational level were not significant predictors of online help-seeking behavior.
Best et al (2016) [9]	Northern Ireland	Modified photo-elicitation techniques were employed within 8 semistructured focus group sessions	High school students, males aged 14-15 years (N=56)	• The *pathways to online help seeking* conceptual model.<br>• Online informal help-seeking pathways increased opportunities for support but included loss of control and anonymity.<br>• Participants have difficulty accessing formal online pathways due to lack of familiarity.
Birnbaum et al (2017) [47]	United States	Pathways to Care for Psychosis Questionnaire (PCP-Q); the PCP-Q consists of approximately 70 open-ended and	Participants recruited from clinical settings, aged 12-21 years (N=80; males =39,	• A third of the sample used the internet as their primary source for obtaining mental health-related information, especially during the emergence of symptoms.<br>• The online environment is clearly a powerful resource regularly used by the vast majority of young people during prolonged periods of untreated illness.

		multiple-choice questions.	females =41)	
Bradford and Rickwood (2014) [35]	Australia	Self-report questionnaire	High school students (females =139; males =92)	• Reason for preference of online support services included the anonymity of the internet; information was easily accessible, and that there are often people in chat rooms who have been through a similar experience.<br>• Adolescents are more likely to not seek help at all.
Burns et al (2016) [13]	Australia	Telephone interview	Young people aged 16-25 years (males =700; females =700)	• Young people who reported moderate to very high levels of psychological distress were more likely to use the internet to seek information about a mental health and alcohol or substance use problem than those with low psychological distress.<br>• Young females were significantly more likely than males to use the internet for accessing health information.<br>• Young people with moderate to very high levels of psychological distress were more likely to use the internet after 11 pm than those with low levels of psychological distress.<br>• The internet is a place where young people spend time, connect with others, and seek help for their problems.

Burns et al (2010) [48]	Australia	Telephone interview	Young people aged 12-25 years (n=2000, females =1000, males =1000)	• Young people reported searching the internet for information about physical or mental health problems regardless of whether they had a problem themselves.<br>• Less than half sourced information from mental health websites, while 50.7% sourced information from other websites, including government websites, online encyclopedias, medical journals, pharmaceutical information, research databases, and tertiary education websites.<br>• Forums, bulletin boards, and discussion or support groups were not common sources of information.
Collin et al (2011) [12]	Australia	Website statistics, user profiling survey, user-generated content, and interview and focus groups	—[1]	The ReachOut website appears to be acting as a gateway service by assisting a young person to identify that they have a problem that warrants help and then supporting the young person to access appropriate treatment in a timely manner.
Ellis et al (2012) [26]	Australia	Online survey: technology use;	Young people aged 16-24 years	• More than half of all males and two-thirds of psychologically distressed males used the internet for help seeking.

[1] Data not available

		attitudes and behaviors; psychological distress (K6), technology and mental health	(N=1038, females =552, males =486	• Significantly more females than males had talked about their problems online.<br>• Majority of the entire sample had sought help for their problems online.
Ellis et al (2013) [25]	Australia	Online survey and focus groups	National online survey of 486 males (aged 16-24 years) and 17 focus groups involving 118 males (aged 16-24)	• Age was a significant predictor for seeking help online, with younger males being more likely to have talked about their problems online than older males.<br>• Young men tend to resist encouraging a friend to seek help but at the same time would require prompting from a close friend to start seeking help.
Feng and Campbell (2011) [27]	Australia	Online survey: demographic items, use of e-mental health information and resources, Eysenck Personality	First year university students (N=176, females =119, males =57)	• Almost half of participants (44%) reported they had used the internet at some point in time to learn about personal feelings of anxiety, sadness, or confusion.<br>• No participants reported that the online resources *made things worse* or *a little worse*.<br>• The sample had a preference for text-based search engines and information sites.

		Questionnaire		• Making e-mental health resources available does not necessarily result in their use.
Frost and Casey (2016) [29]	Australia	Online survey: psychological distress (K10); self-injurious behaviors (The Self-Harm Behavior Questionnaire [SHBQ]); suicidal ideation (SBQ-R); help-seeking intentions (GHSQ)	Young people reporting a history of self-injury (N= 679) were identified as part of larger study (N=1463) exploring help seeking (females =513, males =71)	• Online help seekers were significantly younger than those who did not seek help online in relation to self-injury.<br>• Young people who sought help online in relation to self-injurious behavior were more distressed and had higher levels of suicidal ideation.<br>• Those who had gone online looking for support in relation to self-injury reported a belief that there is more support available to them online.<br>• Online help seekers had significantly higher psychological distress, suicidal ideation, and self-injurious behavior.<br>• Young people who sought help online in relation to self-injurious behavior were less likely to have disclosed their self-injurious behavior to anyone.
Frost et al (2016) [28]	Australia	Online survey	A sample of 1463 remained, with 679 of these participants reporting a history of self-injury.	• Online help seekers were significantly more distressed, suicidal, and had a greater degree of self-injury compared to those who did not seek help online.<br>• Access related to the need for 24×7, free service with instant access and real-time support, from anywhere.

			The qualitative question of relevance in the current paper was completed by 457 (females =399)	• A significantly higher proportion of individuals who had previously been online to seek help in relation to self-injurious behavior endorsed the importance of reduced isolation.<br>• Services delivered online may need to incorporate contact with professionals and peers to be most effective.
Greidanus and Everall (2010) [44]	Canada	Analysis of records created on an online community message board	—[2]	• In all cases, adolescents described their experiences of being suicidal as the result of life stressors and within the context of other life events.<br>• Responding member's messages contained all of the elements that would be included by trained volunteers. The most common of these being empathy, empowerment, and *we care/keep posting*.<br>• Several of the help seekers began asking for support from other members and later began writing to support others.
Haner and Pepler (2016) [40]	Canada	Comparative study: Counselling Client Questionnaire 2 +	232 phone clients (females =169, males =53) and 230	• There was a larger proportion of non-heterosexual participants in the chat sample than in the phone sample.<br>• A significantly larger proportion of chatters sought help for weighty

[2] Data not available

		Objective Appraisal of Risk Level	Live Chat clients (females =201, males =24)	topics (suicidality and mental illness) than callers.<br>• Online chatters were significantly more likely to have spoken to someone perceived as a helping professional such as counsellors or therapists but less likely than callers to have ever had contact with a formal mental health service.<br>• Mental health struggles of Live Chat clients were more frequent and more severe than those of callers in general.
Havas et al (2011) [42]	The Netherla nds	Focus group interviews	High school students aged 12-19 years (females =55, males =51)	• Participants made use of the internet to search for help for mental health problems.<br>• An ideal website would include information, self-tests, and anonymous help.<br>• Layout and design preferences varied and were related to educational level.
Horgan and Sweeney (2010) [30]	Ireland	Online survey: used the internet, including frequency, use of search engines, use of social networking	University students aged 18-24 years (N=922, females =552, males =333)	• In total, 30.8% of participants used the internet for mental health information.<br>• It was suggested that a basic layout with access to chat rooms and a good search facility would be very important. The website should include links to self-help groups, and the website should not be for profit.

		sites, and information on how they use the internet to search for general health information.		• In all, 20.6% of the sample reported that they would prefer internet interventions to face-to-face support.
Kauer et al (2017) [39]	Australi a	Feasibility study	Young people aged 18-25 years (N=51, females =39, males =12)	One of the benefits reported by participants in the qualitative responses was the immediate increase in positive affect after seeking help using the tool.
Mar et al (2014) [43]	Canada	Qualitative interview	Young people aged between 16 and 24 years (N=23, females =22, males =1)	• Participants highlighted the importance of the availability of direct support, for example, peer support, counsellors, and doctors. Chat was seen as the most effective form of communication.<br>• It was important for users to remain anonymous while accessing a service online.<br>• Participants used the internet as a tool to seek help and search for information related to feelings of depression and anxiety.<br>• Users stated that online tools should be simple, look authentic, be easy to access, and be easy to navigate.

				• Having a community was a very important feature for participants.
Mars et al (2015) [36]	United Kingdom	Quantitative	The questionnaire was sent to 8525 participants, of whom 4110 (48.2%) responded and 3946 (46.3%) provided data on their suicide/self-harm-related internet use and previous self-harm	• A greater proportion of participants had accessed sites offering help, advice, or support than accessing sites offering information on how to hurt or kill yourself.<br>• The risk of accessing these potentially harmful sites was considerably elevated among those with suicidal thoughts.<br>• The findings suggest that those who report suicide/self-harm-related internet use were actually more likely to have sought professional help for self-harm/suicidal thoughts than those without.<br>• Females were more likely to report suicide/self-harm-related internet use than males in the study.
Mok et al (2016) [45]	Australia	Online survey: Suicide Behaviors Questionnaire-Revised; Patient Health Questionnai	Young people aged 18-24 years (N=205, females =156, males =45)	• Suicide-related users reported significantly higher levels of social anxiety and lifetime suicidal ideation.<br>• Those who reported higher levels of suicidal ideation and those who reported a higher likelihood of future suicide were more likely to use the internet for suicide-related reasons.

		re; Social Interaction Anxiety Scale; General Help-Seeking Questionnaire; Multidimensional scale of perceived social support; suicide-related internet use; barriers to help seeking		• Suicide-related users are significantly more likely to prefer online help-seeking sources for future help-seeking intentions.
O'Dea and Campbell (2011) [37]	Australia	Cross-sectional survey	High school students (N=85, mean age =14.7 years, n=46 females)	• In total, 53% of the sample had made use of the internet to search for mental health support.<br>• Participants believed that social networking sites could help with mental health–related problems.
Rickwood et al (2015) [41]	Australia	Comparative study: users accessing	Young people aged 12-25 years	• Self-initiation plays a greater role in online help seeking than for offline help seeking.

		face-to-face Headspace and online e-Headspace	(females =48.7%, males =51.3%)	• This was evident for younger age groups too, with two-thirds of the early adolescent age group reporting that they themselves were the main influence to use e-Headspace.<br>• Friend influence appeared to be strongest for the youngest girls within the online environment, and then declined with age. For boys, it peaked online in mid-adolescence and then declined.<br>• The online environment is distinct from traditional in-person mental health care, and this is the environment that young people are navigating on their own from an early age.
Ruppel and McKinley (2015) [31]	United States	Online survey focused on the following measures: overall mental health; social support; social anxiety; usefulness of online resources; previous	University students (N=443, females =297, males =146)	• Individuals who have little social support have a limited view of what resources are available to them and be less likely to take advantage of online mental health resources when necessary.<br>• Participants with higher social support see benefits to online mental health resources but do not necessarily use these sources.

		use of online mental health resources		
Sanci et al (2017) [38]	Australia	Randomized controlled trial	Young people aged 18-25 years	An online referral tool such as Link is useful and provides a positive help-seeking experience.
Spears et al (2015) [32]	Australia	Online survey	Young people aged 12-18 years (N=2338)	• Young people in the cyberbully victim category, in particular, reported poorer mental health, lower levels of social connectedness, and greater stress, anxiety, and depression.<br>• This group was also more likely to access the internet after 11 pm; yet by contrast, they are highly unlikely to access help online, with a quarter seeking help firstly from friends.<br>• Online help sources need closer alignment with the needs of young people.<br>• More needs to be done to promote available online help sources and to better understand why some groups of young people do not seek help online, especially, if they are online late at night when parents and friends may not be available to help.
Wetterlin et al (2014) [33]	Canada	Online survey: (1) demographi	Young people aged 17-	• Most participants indicated that they had used the internet to seek

		cs, (2) mental health literacy, (3) online and offline use of mental health resources, (4) online and offline opinions about current mental health resources, and (5) preferences regarding potential components of a mental health website	24 years (N=521, females =399, males =121)	information or help for the feelings they were experiencing.<br>• The majority of participants (83.9%) listed contact with an online professional (eg, therapist or coach) as either somewhat important or very important.<br>• Professional, peer support, friend involvement, and family involvement as important features of a mental health–related website.<br>• A mental health website would need to have information from credible and trustworthy sources, including descriptions of interventions and treatments.

Table 2. Findings identified in studies: How do young people seek help online?

Author (year)	Findings related to how young people seek help online
Best et al (2016) [22]	• In total, 57% of the sample used a text-based search engine to locate information.<br>• Overall, 48.9% used social media to locate mental health information.

	• Less than quarter of the sample would access through a government website, although they value mental health services run by trained professionals. However, these need to be available 24 hours a day.
Birnbaum et al (2017) [47]	• Participants with non-psychotic mood disorders (NPMD) were primarily interested in obtaining information on how to stop symptoms.<br>• Participants with Psychotic Spectrum Disorders (PSD) were more commonly interested in what caused their symptoms.<br>• Young people are actively reaching out over social media and other online resources before they access professional care.<br>• Young people showed an interest in obtaining help/advice from professionals via social media.
Burns et al (2010) [48]	• Young who are highly distressed were accessing the internet after 11 pm at night.<br>• A total of 21.4% of 12-17-year-olds and 93.3% of the sample used a search engine such as Google or Yahoo to find information rather than accessing a specific website.<br>• In all, 33.9% of 18-25-year-olds reported that they had specifically searched the internet for mental health concerns.<br>• Less than half, that is, 44.4% sourced information from mental health websites, whereas 50.7% sourced information from other websites.
Ellis et al (2013) [25]	Age, however, was a significant predictor for websites and church leader, with younger males being more likely to recommend websites than older males.
Feng and Campbell (2011) [27]	• Overall, 44% reported they had used the internet at some point in time to learn about personal feelings of anxiety, sadness, or confusion.<br>• Participant usage rates of online resources for the purpose of coping with feelings were distributed as follows: (1) 42% used text-based search engines, (2) 26% used informational sites, (c) 22% used Facebook or Myspace profiles, and (4) 11% used online support groups and discussion boards.

Frost et al (2016) [28]	• The most popular suggestion was for "an online service with direct links to professionals in real time via instant messaging," which was endorsed by 54.1% of the young people. Other highly endorsed online sources were peer support such as online forums and chat rooms (48.6%), online self-help programs (43.5%), and information provided in text or fact sheets.<br>• A total of 13.8% the sample reported a desire for information online that would help them to immediately talk to family, friends, or a professional about their self-injurious behavior.<br>• In all, 45.7% of young people indicated a desire for advice or help and support when seeking help online.<br>• Many young people specifically identified a need for information about self-injury, research, statistics, and fact sheets and highlighted the importance of relevance and reliability of such information.
Greidanus and Everall (2010) [44]	• The roles of *help seeker* and *help provider* are dynamic, meaning that a single person may fill a role at different times throughout the message boards. After posting messages seeking help, help seekers wrote messages to encourage each other and a community was developed that included help seekers, help providers, and trained volunteers who monitored the posts.<br>• Each help seeker began the thread by posting a message expressing personal distress that was often related to suicidal feelings or concern that someone close to them was suicidal.
Haner and Pepler (2016) [40]	When young people are extremely distressed, they seem to access Live Chat options.
Horgan and Sweeney (2010) [30]	1. A total of 30.8% of participants used the internet for mental health information.<br>2. The main information searched for was information on depression, information for course work, general information on mental health problems, and information on specific problems they themselves or family or friends experienced.

Mars et al (2015) [36]	• A greater proportion of individuals had accessed sites offering help, advice, or support than had accessed sites offering information on how to hurt or kill yourself.<br>• Almost half of those with suicidal self-harm had searched for information about suicide and self-harm and 30.2% had used the internet to discuss suicidal feeling.<br>• Many of the sample had looked for information about self-harm or suicide using a search engine or used the internet to discuss self-harm or suicidal feelings.<br>• Suicide/self-harm-related internet use was particularly prevalent among those who had self-harmed with suicidal intent (70%).
Mar et al (2014) [43]	The majority of the sample perceived it to be common and obvious to google *depression symptoms* and/or *depression treatment*, and searched for these phrases themselves.
Wetterlin et al (2014) [33]	• A total of 52.4% of participants indicated that when using the internet for mental health information-seeking purposes, they were looking for information about symptoms and 47.4% were looking for treatment options.<br>• Most (82.9%) participants were either *somewhat likely* or *very likely* to use an information-based website with mainly text. Slightly more than half of the participants were *not at all likely* or *somewhat unlikely* to use online interactions such as a group online chat session led by a psychologist (55.3%) and chat rooms/support groups/discussion boards (56.6%).<br>• Only 10.6% said that they had used social media (eg, Facebook and MySpace) to obtain help with problems such as anxiety or depression. –<br>• Does not appear that this sample used social networking sites when looking for help or support for problems such as anxiety or depression.
